# Five genetic variants explain over 70% of hair coat pheomelanin intensity variation in purebred and mixed breed domestic dogs

**DOI:** 10.1371/journal.pone.0250579

**Published:** 2021-05-27

**Authors:** Andrea J. Slavney, Takeshi Kawakami, Meghan K. Jensen, Thomas C. Nelson, Aaron J. Sams, Adam R. Boyko

**Affiliations:** 1 Embark Veterinary, Inc., Boston, Massachusetts, United States of America; 2 Department of Biomedical Sciences, Cornell University College of Veterinary Medicine, Ithaca, New York, United States of America; HudsonAlpha Institute for Biotechnology, UNITED STATES

## Abstract

In mammals, the pigment molecule pheomelanin confers red and yellow color to hair, and the intensity of this coloration is caused by variation in the amount of pheomelanin. Domestic dogs exhibit a wide range of pheomelanin intensity, ranging from the white coat of the Samoyed to the deep red coat of the Irish Setter. While several genetic variants have been associated with specific coat intensity phenotypes in certain dog breeds, they do not explain the majority of phenotypic variation across breeds. In order to gain further insight into the extent of multigenicity and epistatic interactions underlying coat pheomelanin intensity in dogs, we leveraged a large dataset obtained via a direct-to-consumer canine genetic testing service. This consisted of genome-wide single nucleotide polymorphism (SNP) genotype data and owner-provided photos for 3,057 pheomelanic mixed breed and purebred dogs from 63 breeds and varieties spanning the full range of canine coat pheomelanin intensity. We first performed a genome-wide association study (GWAS) on 2,149 of these dogs to search for additional genetic variants that underlie intensity variation. GWAS identified five loci significantly associated with intensity, of which two (CFA15 29.8 Mb and CFA20 55.8 Mb) replicate previous findings and three (CFA2 74.7 Mb, CFA18 12.9 Mb, CFA21 10.9 Mb) have not previously been reported. In order to assess the combined predictive power of these loci across dog breeds, we used our GWAS data set to fit a linear model, which explained over 70% of variation in coat pheomelanin intensity in an independent validation dataset of 908 dogs. These results introduce three novel pheomelanin intensity loci, and further demonstrate the multigenic nature of coat pheomelanin intensity determination in domestic dogs.

## Introduction

For thousands of years, humans have selectively bred domestic dogs for desired physical and behavioral phenotypes, including a wide variety of coat colors and patterns [1, 2]. For example, historical writings indicate that shepherds from as early as the first century AD preferred white-colored herding and livestock guardian dogs because this coloration allowed them to quickly distinguish their dogs from wolves [3], while some modern sporting breeds such as Chesapeake Bay Retrievers have been selectively bred to have dark to light brown coats “colored to match their working environment” [4]. Indeed, nearly all modern breed standards published by various kennel clubs provide detailed specifications on coloration. Genetic mapping studies have identified several key genes that account for much of the coat color and patterning variation across domestic dog breeds [5–16], but the genetic bases of some common phenotypes remain unclear. An overview of canine pigmentation genetics is provided in [17].

All canine coat colors and patterns result from varied expression of two pigment molecules: eumelanin, which is black or brown, and pheomelanin which is reddish-yellow. Most canids have coats containing a mixture of hairs expressing eumelanin, pheomelanin, or both, but many domestic dogs have coats in which only pheomelanin is expressed. These “pheomelanic” coats result from mutations in and around one of two genes that regulate switching between eumelanin and pheomelanin synthesis in hair follicle melanocytes: melanocortin 1 receptor (*MC1R*, known as the “E locus”) and agouti signaling protein (*ASIP*, known as the “A locus”) [14]. At least four different recessive mutations in and around the *MC1R* gene inhibit the synthesis of eumelanin in hair follicle melanocytes, resulting in a solid “recessive red” coat containing only pheomelanin [5–7, 17, 18]. A completely or mostly red coat can also result from carrying a dominant *ASIP* variant (A^y^), which produces “sable” coats with varying amounts of black/brown hairs concentrated around the dorsal midline, and pheomelanic hairs across the rest of the body [8, 15].

The intensity of pheomelanic coloration varies widely across and within breeds that are fixed for recessive red or sable coats. For example, Irish Setters have consistently deep red coats, while Soft-coated Wheaten Terriers have coats that vary from cream to tan. Additionally, many breeds with solid white or cream coats have been shown to be recessive red, including Bichon Frisé, Samoyed, West Highland White terrier, and White German Shepherd [5, 19]. Over decades of research, uncovering the genetic basis of pheomelanin intensity variation in dogs has proven to be unexpectedly challenging. It was originally hypothesized that extreme pheomelanin dilution in pheomelanic dogs–resulting in a white or cream colored coat–was primarily controlled by a single locus [20, 21], as it is in several other mammalian species [22–31]. However, it is increasingly apparent that even this one extreme of coat pheomelanin intensity is a multigenic trait across, and perhaps within, dog breeds.

Three recent studies have identified several genetic variants that are able to explain some coat pheomelanin intensity variation in certain breeds. The first study identified two variants in and upstream of the *MC1R* gene that are highly predictive of extreme pheomelanin dilution in recessive red Siberian Huskies and Australian Cattle Dogs [18], but did not investigate how these variants affect coat pheomelanin intensity in other breeds. A second study identified a missense mutation in the major facilitator superfamily domain containing 12 gene (*MFSD12*) that is associated with extreme pheomelanin dilution in a wide variety of breeds [19]. However, dogs that were homozygous for the mutation still showed variation in pheomelanin dilution within some breeds, suggesting that pheomelanin dilution is a multigenic trait both across and within breeds. Similarly, a third study identified a copy number variant upstream of the KIT ligand gene (*KITLG*) that was predictive of red intensity in Nova Scotia Duck Tolling Retriever and Poodle [32], but not in two of the most common (in the United States [33]) and phenotypically variable breeds: Golden Retriever and Labrador Retriever. In this study, our aim was to increase understanding of the genetic underpinnings of coat pheomelanin intensity variation in dogs by testing whether there are additional loci that affect intensity across dog breeds, and investigating how these loci might interact. We achieved this by performing a genome-wide association study (GWAS), which identified five genomic regions that are significantly associated with coat pheomelanin intensity, and showing that these loci are able to explain approximately 70% of variation in coat pheomelanin intensity in mixed breed and purebred dogs.

## Materials and methods

### Ethics statement

Participating dogs were part of the Embark Veterinary, Inc. customer base. Owners provided informed consent to use their dogs’ data in scientific research by agreeing the following statement: “I want this dog’s data to contribute to medical and scientific research”. Ethical approval was not required as non-invasive methods for genotype or phenotype collection were used (buccal swabbing and photographing, respectively). Dogs were never handled directly by researchers. Owners were given the opportunity to opt-out of the study at any time during data collection. The discovery and validation cohorts were selected from data available collected between October 2018 and June 2020. All published data have been de-identified of all Personal Information as detailed in Embark’s privacy policy (embarkvet.com/privacy-policy/).

### Genotype and phenotype data collection

Cheek cell samples were collected by dog owners with buccal swabs, and DNA was extracted by Illumina, Inc. and genotyped at 221,188 biallelic autosomal and X chromosome markers on the Embark Veterinary custom Illumina CanineHD SNP array [34, 35]. Dogs that had been genotyped between October 2018 and June 2020 were filtered to those that 1) had owner consent to use of their genetic data and owner-reported data for research, 2) had at least one owner-provided photo, 3) had owner reported breed assignments, and 4) were genetically “recessive red” (e/e at the E locus [6]) or “sable” (ky/ky at the K locus and Ay/Ay, At, Aw, or a at the A locus [8]) per their array genotypes. Of the 3,596 dogs that met these four criteria, 72 were excluded from further analysis due to discrepancy between genetic analysis and owner-reported breed, leaving 3,524 to be phenotyped. Breed assignments and genotypes at the E, K, and A loci for the 3,057 dogs that passed subsequent quality control steps are available in S1 File.

### Phenotyping

**Fig 1:**
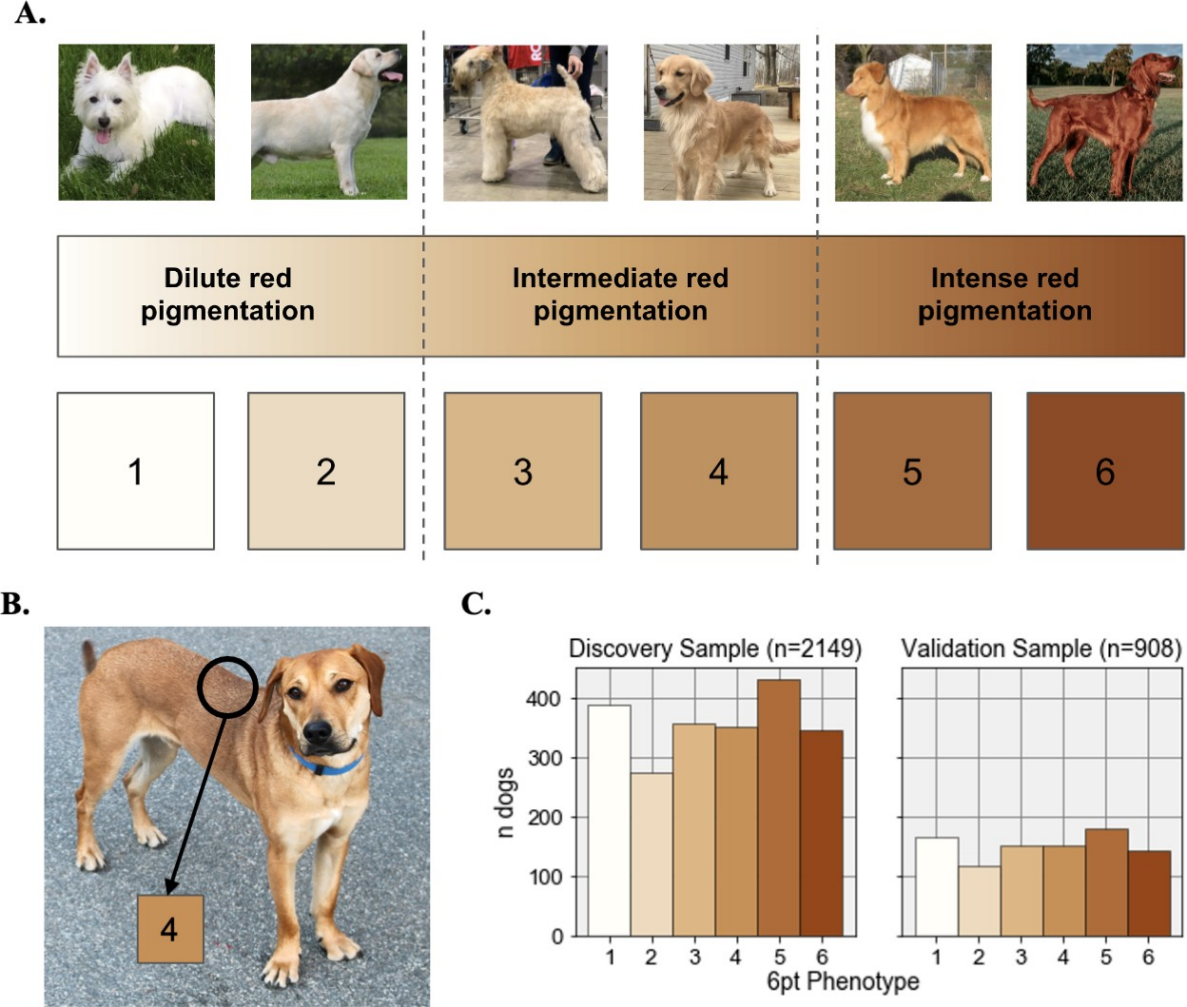
The six point coat pheomelanin intensity scale. **A.** Photos of six purebred dogs that exhibit the full range of coat pheomelanin intensity in canids are shown above a continuous color scale and numbered swatches showing the color of each of the six phenotype values used in this study. From left to right, the breeds of the dogs in these photos are: West Highland White Terrier, Yellow Labrador Retriever, Soft-coated Wheaten Terrier, Golden Retriever, Nova Scotia Duck-Tolling Retriever, Irish Setter. All six dogs pictured were part of the study sample. **B.** An example of a dog that displays “countershading”. The black circle indicates the part of the photo that was used to assign this dog’s phenotype (4 on the six point color scale), which in this case was the mid back. **C.** Histograms showing the number of dogs with each phenotype value in the discovery and validation samples.

To develop a color scale for visual phenotyping, we selected three shades (cream, tan, and red) that encompass the range of coat pheomelanin intensity phenotypes in domestic dogs and obtained their hexadecimal values (#FFFEF9, #D3A467, and #93471A). We then used the Matplotlib [36] LinearSegmentedColormap and Normalize functions to obtain six equally spaced hexadecimal values spanning the range of values defined by these three colors. The six point coat color scale (Fig 1A) consists of the colors encoded by these hexadecimal values: #FFFEF9 (1), #EDDABF (2), #DCB684 (3), #C69158 (4), #AD6C39 (5), and #93471A (6).

To assign coat color phenotypes to dogs, a single scientist visually evaluated owner-provided photos and assigned each dog to one of the six levels in the coat color scale or excluded it from further analysis. To account for red countershading—meaning darker red hair along the back, ears, and the tip of the tail in some breeds (Fig 1B)—all dogs were typed based on their coat color at the top of the mid back, or if the back could not be clearly seen, the top of the head. The pheomelanin intensity phenotype could not be confidently typed based on available photos for 215 dogs (due to poor photo quality, positioning of the dog in the photo, multiple dogs shown in the same photo, or lack of red hair on the head or shoulders due to coat patterning) and these were excluded from further analyses.

At this point, our sample contained an excess of purebred dogs from breeds that are fixed for cream coats compared to breeds that are fixed for red coats. In order to achieve a better balance between these two extremes, we used concordant owner-reported and genetically-determined breed assignments to identify an additional 197 genetically pheomelanic, purebred dogs with no owner-provided photo that belonged to breeds that are fixed for red coats (5 or 6 on our phenotype scale). These dogs were assigned the most common six-point phenotype value in their breed across the rest of the sample. The dogs phenotyped in this manner consisted of 21 Brittanys, 2 Ibizan Hounds, 4 Irish Setters, 5 Irish Red and White Setters, 8 Redbone Coonhounds, 138 Rhodesian Ridgebacks, 16 Vizslas, and 3 Welsh Springer Spaniels (the 129 of these dogs that passed subsequent filtering steps are indicated in S1 File). Including these, our dataset consisted of 3,501 dogs with confident phenotype and breed assignments.

To assess phenotyping consistency, 350 dogs with photos were randomly selected (from the final set of 3,057 dogs that passed subsequent filtering) using the pandas DataFrame.sample() method [37] and re-phenotyped on the six point scale by the same scientist who performed the original phenotyping. The concordance between the original and new phenotypes was 97%, and 100% of dogs had a new phenotype value that was within 1 point of their original phenotype value (S1 Fig in S1 Appendix, S1 File).

### Genotype data filtering

PLINK 1.9 [38] was used to remove array markers with >5% missingness (n = 16,617) and dogs with >3% missingness (n = 3) across the remaining markers. We then removed 441 close relatives from the remaining dogs by identifying pairs of dogs with pi_hat ≥ 0.45 (calculated using PLINK 1.9’s—genome utility) and dropping the dog with the higher genome-wide missingness in each pair from the dataset. After these steps, the total genotyping rate was 99.9% across 204,571 markers in 3,057 dogs from 63 different breeds and varieties. These data are available in S1 File.

### Discovery and validation data partitioning

We grouped the 3,057 dogs according to their breed, subset each breed by six point phenotype value, and split each phenotype group randomly 70:30 into the discovery and validation datasets using the pandas DataFrame.sample() method [37]. As a result, the breed ancestry (S1 Table) and phenotype (Fig 1C) distributions were highly similar between our discovery and validation datasets, with both datasets having at least one individual from each of the 63 breeds. The discovery dataset partitions were combined (n = 2,149) and used as input to the discovery GWAS, then used as a training dataset to define marker weights in the predictive models. The validation data partitions were combined (n = 908) and used to assess the accuracy of the predictive model (see “Predictive models for coat pheomelanin intensity” below).

### Genome-wide association

To identify genomic regions associated with pheomelanin intensity variation, we encoded coat color as both a case-control (cream versus red) and quantitative trait (six point scale) and applied a multivariate linear mixed model implemented in GEMMA v.0.98 [39] to our discovery dataset. To further account for confounding effects of shared ancestry among dogs of the same or closely related breeds, kinship matrices were constructed from array genotypes using the GEMMA -gk command and used as a random effect in the model for each GWAS run. Setting GEMMA’s -miss and -maf values to 0.05 and 0.001 led to 16,343 markers being excluded from analysis, for a total of 188,288 markers in 2,149 dogs. The association result files generated by GEMMA are available in S1 File. In all GWAS, we used the Bonferroni correction with an alpha of 0.05 as a threshold for considering a SNP to be significant at the genome-wide level.

An initial GWAS run showed marginally significant associations in the *MC1R* and *RSPO2* genes on canine chromosome (CFA) 5 and CFA13, respectively (S2 Table, S2 Fig in S1 Appendix). The top markers at these loci—CFA5: 63,694,334 and CFA13: 8,611,728, respectively—are in fact known causal mutations for recessive red (*MC1R “*e” [6]), and tightly linked to the indel causing “furnishings” [40], which refers to longer hair along on the snout as seen in breeds such as West Highland White Terrier and Bichon Frisé. Several breeds that have lower intensity phenotype values are fixed for the recessive red genotype at *MC1R* and/or have a high frequency of the “furnished” (“F”) allele at *RSPO2* (S1 Table, S1 File). As a result, we determined that these signals were likely driven by differences across phenotype groups that are not directly related to coat pheomelanin intensity. To account for this, we included dogs’ genotypes at the top CFA5 and 13 markers as covariates in our GWAS models which eliminated these association signals (S2 Table, S2 Fig in S1 Appendix). We discuss the association results produced by the GWAS models including these covariates in the Results.

Due to the difficulty of obtaining appropriate hair samples for the thousands of dogs in our sample from individual owners, we were not able to experimentally measure the amount of pheomelanin in dogs’ hair coats (as done in [32]). Because of this, we could not test the assumption that our phenotype values were truly quantitative. To account for the possibility that treating our phenotype values as quantitative might create spurious associations, we performed a case-control GWAS contrasting cream (phenotype value 1 or 2) and red (phenotype value 5 or 6) dogs. The case-control and quantitative GWAS detected the same set of top markers (S2 Fig in S1 Appendix, S2 Table), so we focus on the quantitative GWAS results in the remainder of this manuscript. All genotype, phenotype, and covariate data necessary to replicate all GWAS results are available in S1 File.

### Analysis of public whole genome sequencing data

Raw whole genome paired-end short read sequencing datasets were downloaded as fastq files from the Sequence Read Archive [41] and aligned to the canFam3.1 reference genome using the BWA-MEM algorithm in BWA version 0.7.17 [42]. The mapped reads were filtered and soft-clipped using the Picard Tools version 2.21.4 [43] CleanSam tool, then converted to sorted and indexed.bam files using samtools. Duplicate reads were identified and removed using the Picard Tools MarkDuplicates tool. For regions of interest, the mean depth of sequencing coverage across all autosomes was calculated using the Genome Analysis Toolkit 3 [44] DepthOfCoverage tool, and depth of coverage values in regions of interest were divided by the mean autosomal depth of coverage to obtain normalized depth of coverage values.

To determine which allele at each top GWAS marker was most likely the ancestral allele, we obtained genotypes at these markers across 54 publicly available wild canid whole genome sequencing datasets (1 Dingo, 48 Gray Wolves, 3 Coyotes, 1 Dhole, and 1 Golden Jackal) from a previously published dataset available in the NCBI Sequence Read Archive (SRA) [41, 45]. Genotypes and SRA data accession numbers for these 54 datasets are available in S3 Table. To assess the correlation between a previously discovered copy number variant (CNV) [32] and one of our top GWAS markers, we also downloaded 23 domestic dog whole genome sequencing datasets from SRA and compared their normalized depth of coverage values within the CNV range to their genotypes at the SNP in question. SNP genotypes, normalized read depth within the CNV range, breed, and SRA data accession numbers for these dogs are shown in S4 Fig in S1 Appendix, and are available for download in S5 Table.

### Test for epistatic interactions among GWAS hits

We used the PLINK 1.9 [38]—epistasis tool to test for epistasis among pairs of the top five GWAS variants in the discovery sample. This tool fits a multivariate linear regression model Y = β0 + β1gA + β2gB + β3gAgB for each variant pair (A, B), where Y is the quantitative phenotype value, gA and gB are allele counts, β1 and β2 are the effects sizes of variants A and B, β3 is the effect size of the interaction between A and B, and β0 is a random effect. We considered interactions with a p-value of < 0.05 to be statistically significant.

### Estimation of dominance effects

To evaluate the dominance relationship between the alleles at each of the top GWAS SNPs, we estimated predicted heterozygote phenotype values under complete additivity as the midpoint of the standardized six point phenotype values in the two homozygote classes [46]. We then estimated the dominance effect *d* for each SNP as the difference between the observed and expected mean phenotype values in the heterozygote class. A positive value of *d* is consistent with the red-associated allele being at least partially dominant, and a negative value of *d* is consistent with the red-associated allele being at least partially recessive. We considered *d* to be statistically significant if the 95% confidence interval of the observed heterozygote mean phenotype did not include the additive heterozygote midpoint phenotype. All data used in this analysis are available in S4 Table.

### Predictive models for coat pheomelanin intensity

Using the linear_model module in the Python scikit-learn package version 0.21.3 [47], we fit multivariate linear regression models on the discovery cohort dogs with coat color phenotypes as the dependent variable. In these models, the independent variables were genotype dosage values (coded additively, or with one allele completely dominant to the other) at the five top GWAS markers, as well terms representing their pairwise interactions (i.e. the product of the dosage values at the two individual loci). The coefficients, standard error, t-test values for each independent variable, as well as the y-intercept, adjusted R-squared, and log likelihood values for the best fit model are given in Table 3. These values are also given for all other tested models in S1 File.

## Results

### GWAS identifies five loci associated with coat pheomelanin intensity variation

**Fig 2:**
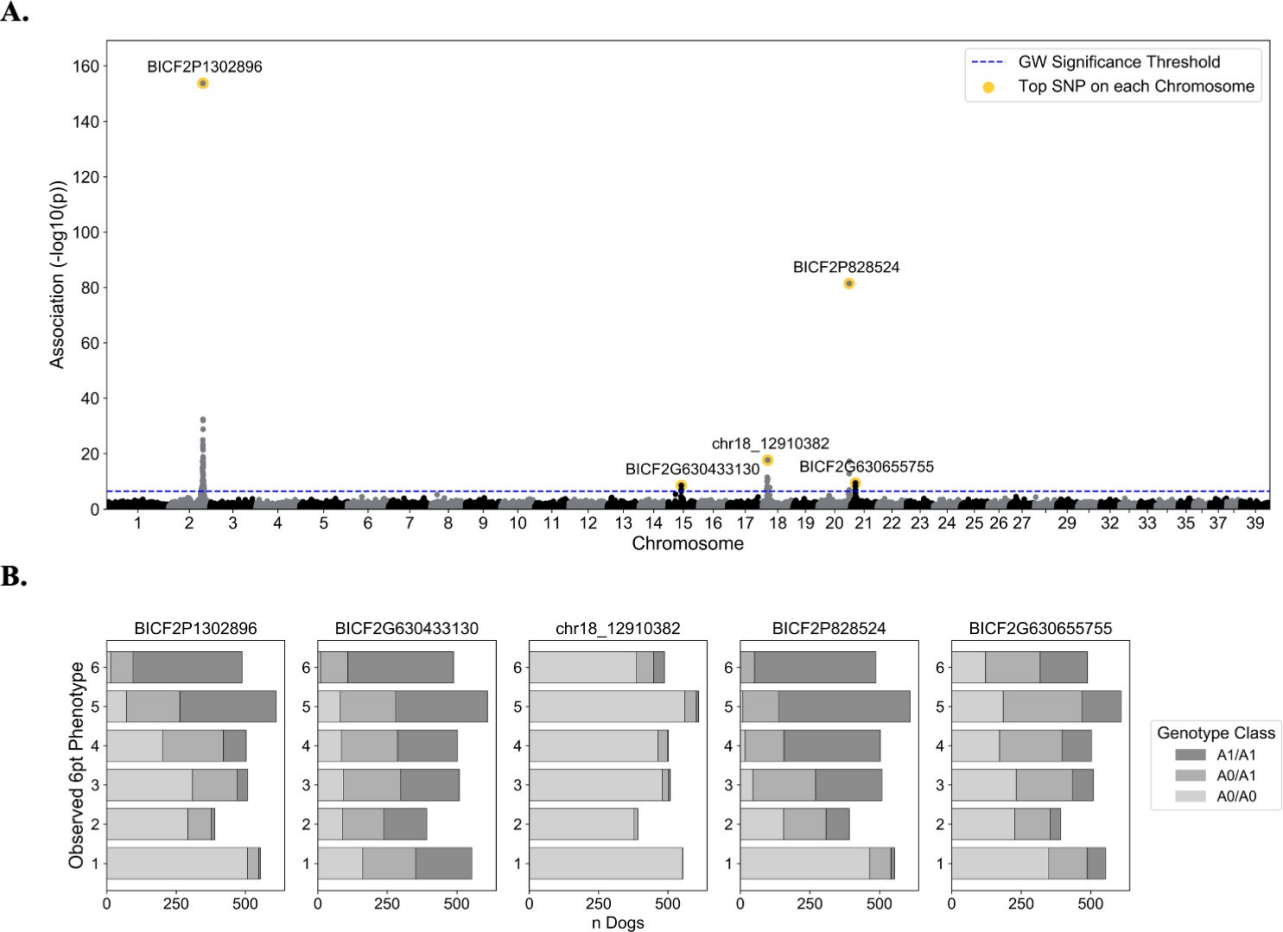
Quantitative coat pheomelanin intensity GWAS results. **A.** GWAS p-values are shown in a Manhattan plot for the autosomes (chromosome 1–38) and the X chromosome (chromosome 39). For each chromosome with one or more genome-wide significant markers, the top marker on the chromosome is highlighted in gold and labeled with its marker ID. The blue dashed line shows the minimum unadjusted -log_10_(p-value) for genome-wide significance using the Bonferroni correction: 6.56. **B.** Bar plots show the number of dogs with each phenotype value (1–6) for each genotype class at each of the top five GWAS markers. The genotype classes are coded according to the dosage of the red-associated alleles at each marker, which are listed in Table 1 as “Allele 1”.

GWAS treating coat pheomelanin intensity phenotypes as a quantitative trait in the discovery dataset identified five significantly associated genomic regions on CFA2, 15, 18, 20, and 21. A total of 88 SNPs passed the Bonferroni correction threshold of 2.73e-7 (6.56 on the -log_10_ scale) (S1 File). The most strongly associated markers in these regions were CFA2: 74,746,906 base pairs (bp) (BICF2P1302896), CFA15: 29,840,789 bp (BICF2G630433130), CFA18: 12,910,382 bp (chr18_12910382), CFA20: 55,850,145 bp (BICF2P828524), and CFA21: 10,864,834 bp (BICF2G630655755) (Fig 2, Table 1).

**Table 1:** Top GWAS markers at five associated loci.

Marker ID	canFam3.1 Pos	Gene	Red Allele, Freq.	Beta, se	-log_10_(Wald’s p-value)	PVE
BICF2P1302896	2: 74,746,906	lincRNA (exonic)	A, 0.42	0.95, 0.03	153.83	0.28
BICF2G630433130	15: 29,840,789	Intergenic	G, 0.66	0.23, 0.04	8.60	0.02
chr18_12910382	18: 12,910,382	*SLC26A4* (exonic)	G, 0.05	0.88, 0.1	17.76	0.04
BICF2P828524	20: 55,850,145	Intergenic	G, 0.65	0.78, 0.04	81.45	0.16
BICF2G630655755	21: 10,864,834	*TYR* (intronic)	A, 0.38	0.23, 0.04	9.51	0.02

Marker IDs, physical position in the canFam3.1 reference genome, gene symbol (if applicable), the red-associated allele and its frequency (Red Allele, Freq.), effect size (Beta) and standard error (se) of the effect size, uncorrected -log_10_(Wald’s p-value), and proportion of variance explained (PVE) for the most significant marker at each of the five associated loci.

The locations of these markers relative to annotated canFam3.1 functional elements in the Ensembl Genes (v95) database [48], as well as r^2^ between genotypes at each top GWAS variant and neighboring variants (i.e. linkage disequilibrium), are shown in S3 Fig in S1 Appendix. The genotypes at the top five GWAS markers in 54 wild canid genomes are available in S3 Table.

### Three novel regions associated with coat pheomelanin intensity

**Fig 3:**
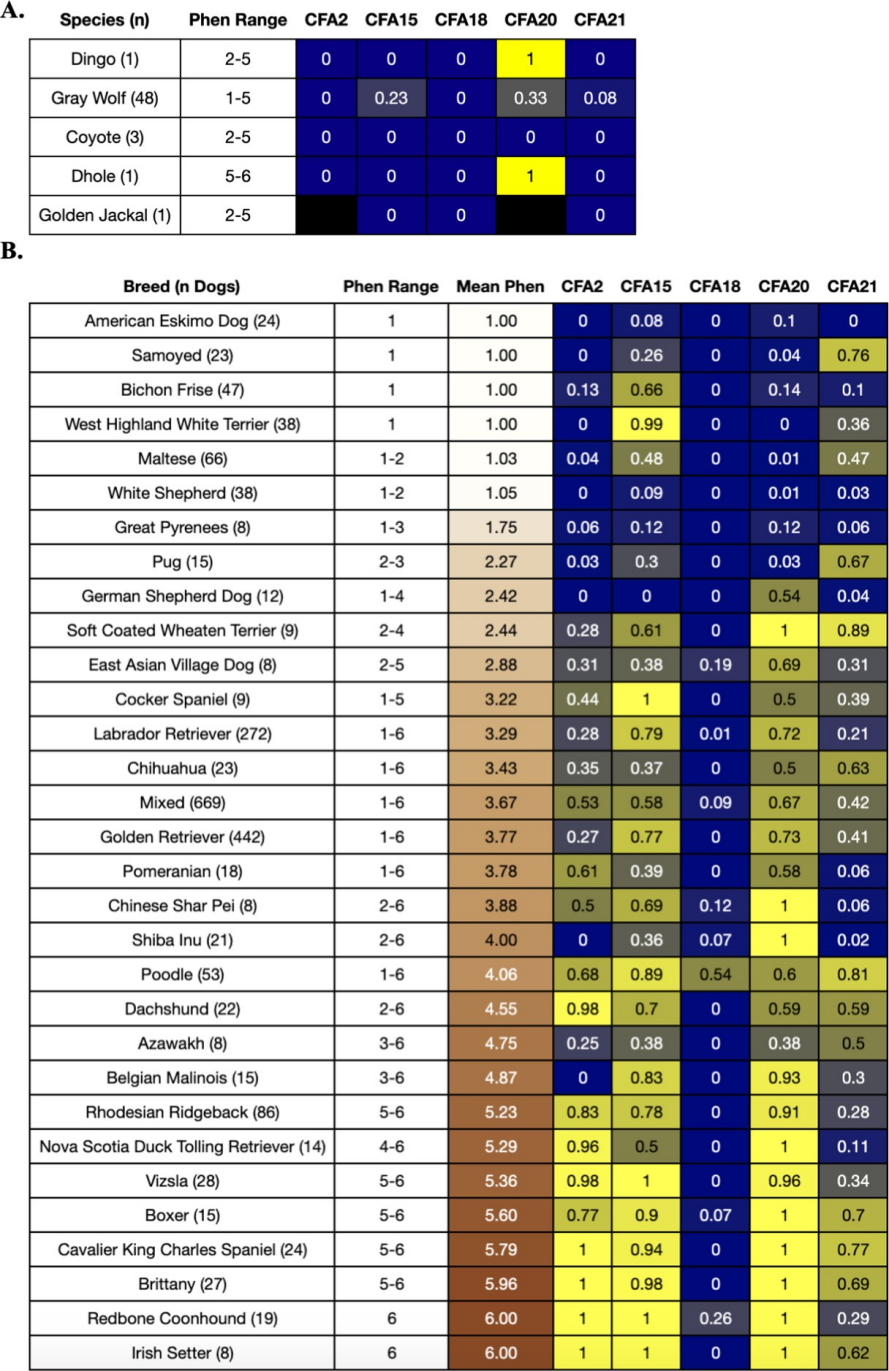
Species and breed allele frequencies at top GWAS markers. Panel **A.** shows the frequencies of the red-associated allele at the top five GWAS markers in 54 public wild canid genomes [45], and panel **B.** shows the same information across 31 breeds with at least 8 individuals in the GWAS sample. Each row shows the breed/species phenotype value range and (for phenotyped dogs, i.e. the dogs in the GWAS sample) the mean phenotype value for each breed, with the mean phenotype value colored by the corresponding coat color. The remaining columns show the breed/species allele frequencies (blue = lower allele frequency, yellow = higher allele frequency, black = no data) of the red-associated alleles at each of the top five GWAS markers, which are labelled according to their chromosome number. Mean phenotype and allele frequency values are colored white or black to improve readability.

To the authors’ knowledge, the CFA2, 18, and 21 associations with coat pheomelanin intensity have not been previously reported. The top CFA2 variant, BICF2P1302896, falls within the second exon of the long intergenic non-coding RNA (lincRNA) ENSCAFG00000042716 at CFA2: 74,744,598–74,747,735 bp (S3 Fig in S1 Appendix). At this marker, the wild canid genomes we examined only carried the cream-associated allele, indicating that the red-associated allele is most likely derived and possibly dog-specific (Fig 3A). The red-associated allele was present in most of the domestic dog breeds we examined, but it was only fixed in breeds with consistently high coat pheomelanin intensity such as Brittany, Redbone Coonhound, and Irish Setter (Fig 3B). The cream-associated allele was fixed in several breeds that are fixed for completely cream coats, including American Eskimo Dog, Samoyed, West Highland White Terrier, and White Shepherd (Fig 3B).

The top CFA18 variant, chr18_12910382, is a missense mutation p.I487M in a conserved residue of the twelfth exon of the solute carrier family 26 member 4 gene (*SLC26A4*) (S3 Fig in S1 Appendix). Like the top CFA2 GWAS marker, the wild canid genomes we examined only carried the cream-associated allele at this marker, indicating that the red-associated allele is most likely derived and possibly dog-specific (Fig 3A).

The top CFA21 variant, BICF2G630655755, falls within the second intron of the tyrosinase gene (*TYR)* (S3 Fig in S1 Appendix). At this marker, only the cream-associated allele was present in Dingo, Coyote, Golden Jackal and Dhole. Although both alleles were present in Gray Wolves, the cream-associated allele is more common and therefore most likely ancestral (Fig 3A). In domestic dogs, both alleles were present in most breeds (Fig 3B).

### Two top associations replicate previous findings

The top CFA15 variant, BICF2G630433130, is located approximately 8 kilobases (kb) downstream of a 6 kb copy number variant (CNV) near the KIT ligand gene (*KITLG*) that was previously associated with variation in coat pheomelanin intensity in Nova Scotia Duck Tolling Retrievers and Poodles (S3 Fig in S1 Appendix) [32], as well as squamous cell carcinoma of the digit in eumelanistic, but not recessive red, Standard Poodles [49]. The red-associated allele at this marker was present at an intermediate frequency (23%) across 48 Gray Wolves, but not in Coyote, Dhole, or Golden Jackal (Fig 3A). Consistent with Weich et al. [32], the red-associated variant segregates at high frequencies in breeds that consistently have high coat pheomelanin intensity but is also segregating at high frequencies in some breeds that are fixed for extreme pheomelanin dilution, such as West Highland White Terrier (Fig 3B).

The top CFA20 variant is the same variant reported in another coat pheomelanin intensity GWAS using over 90 different breeds, which was used to fine map the peak to a nearby missense mutation in the major facilitator superfamily domain containing 12 gene (*MFSD12*) at CFA20: 55,856,000 bp (S3 Fig in S1 Appendix) [19]. We observed that the red-associated allele at BICF2P828524 was segregating at an intermediate frequency in Gray Wolves and carried by the single Dhole and Dingo that we had data for, but absent in 3 Coyotes genomes, making it difficult to infer which allele is ancestral. Consistent with the Hédan et al. [19] study, the red-associated allele was more common across domestic dogs than the cream-associated allele, and while the cream-associated allele was far more common in breeds that are fixed for extreme pheomelanin dilution, it was rarely fixed in those breeds (Fig 3B).

Most of the dogs in our GWAS sample were genotyped prior to the publication of Hédan et al. [19] and Weich et al. [32]. As a result, they were not directly genotyped at CFA20: 55,856,000 bp or the CFA15 CNV upstream of *KITLG*. To evaluate the extent to which our top CFA15 marker is predictive of copy number at the CFA15 CNV, we downloaded publicly available whole genome short-read sequence datasets for 23 dogs of various breeds from the Sequence Read Archive [38], and for each dog, calculated the average read depth across the CNV base pair range and obtained its genotype at BICF2G630433130. The number of red-associated alleles at BICF2G630433130 correlated with a higher mean read depth across the CNV range (Kruskal Wallis test, p-value = 9.99 x 10^−4^; S4 Fig in S1 Appendix), suggesting that the GWAS signal at BICF2G630433130 is likely associated with this CNV.

Of the 2,149 dogs in our discovery dataset, 974 were run on a version of the genotyping array that included both BICF2P828524 and a new marker at CFA20: 55,856,000 bp (these genotypes are included in S1 File). Across these dogs, the overall r^2^ between genotypes at the two markers was 0.77. Thus, we concluded that our GWAS signal at BICF2P828524 is likely primarily or solely driven by the previously identified missense mutation in *MFSD12*.

### Relationship between associated QTL and coat pheomelanin intensity

Within the GWAS sample, several breeds with consistently cream or red coats showed complete fixation of the cream- or red-associated allele (respectively) of at least one marker (Fig 3B). However, no combination of variants was necessary or sufficient to completely explain coat pheomelanin intensity across all breeds.

#### Dominance

**Fig 4:**
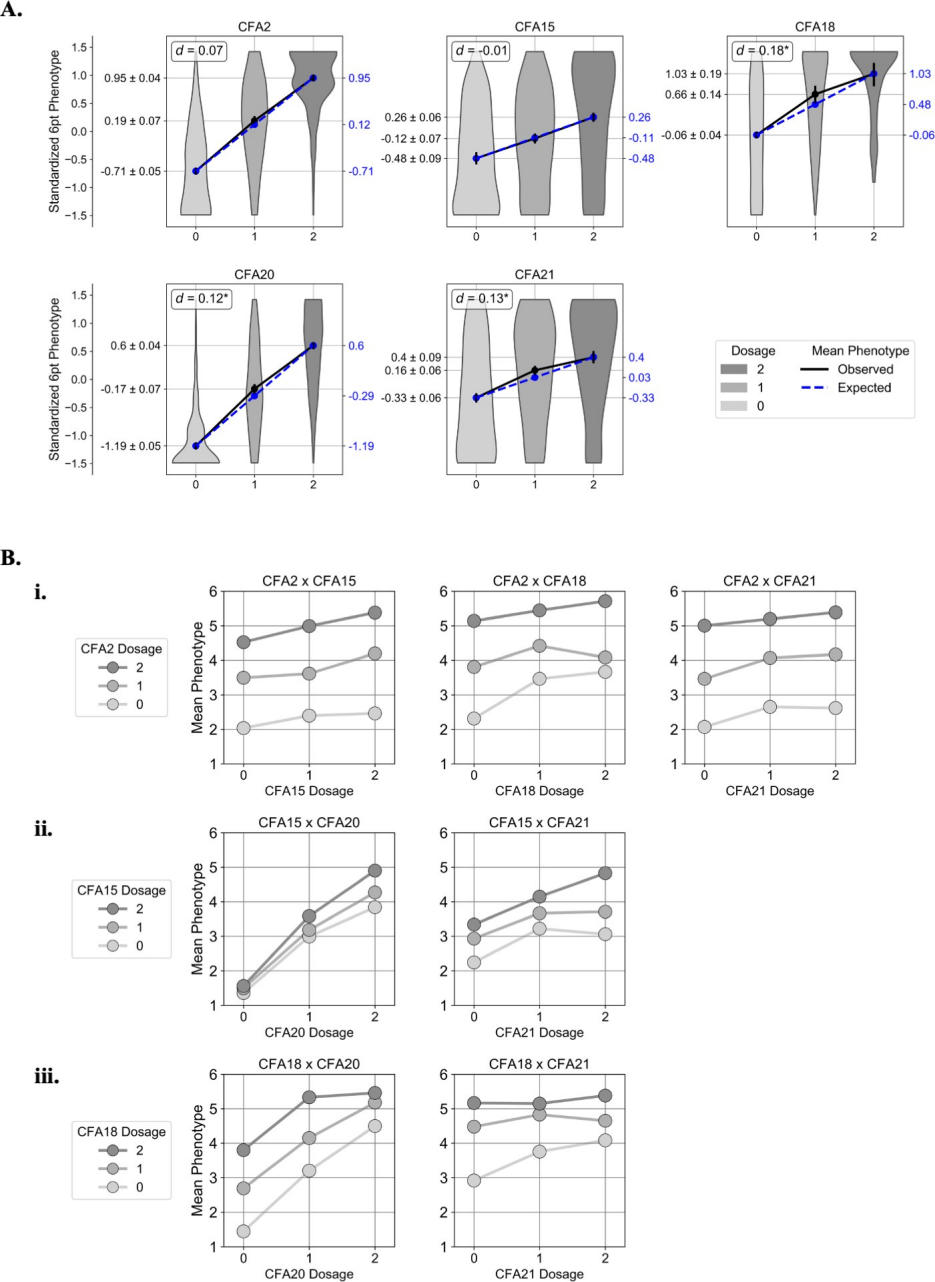
Dominance and epistatic interactions. **A.** For each of the top five GWAS markers, violin plots show the distribution of observed normalized six point phenotype values for each genotype class. The black lines connect the observed means of the three genotype classes, and the blue lines connect the expected means under a perfectly additive model. The estimated dominance coefficient for each marker, *d*, is shown in the upper left hand corner of each plot. An asterisk indicates that the predicted heterozygote class mean phenotype fell outside the 95% confidence interval of the observed heterozygote mean phenotype, which we interpret to mean that *d* is statistically significant. **B.** Scatter plots showing genotype-phenotype interactions at the seven locus pairs that showed statistically significant interaction effects per the epistasis test. In each plot, the “dosage”, i.e. the diploid genotype coded as the number of red-associated alleles, is displayed on the X axis, and the dosage at the other marker is represented by the three lines connecting the points. The Y axis shows the mean six point coat pheomelanin intensity phenotype across dogs with each genotype combination.

For each of the top GWAS SNPs (which we refer to by their chromosome number in the remainder of this manuscript), we estimated the dominance effect *d* as the difference between the observed and expected mean standardized six point phenotype value for the heterozygote class (Methods) (Fig 4A; S4 Table).

We found that the heterozygote mean phenotypes expected under additivity at the top CFA2 and 15 SNPs fell within the 95% confidence intervals of the observed heterozygote mean phenotypes, suggesting that these loci behave in a mostly additive manner. At the top CFA18, 20, and 21 SNPs, the mean heterozygote phenotypes were significantly higher than the additive expectations, suggesting that the red-associated alleles at these loci are at least partially dominant to the cream-associated alleles.

#### Epistasis

When pairwise tests for epistatic interaction were applied to the top five GWAS variants, seven pairs of variants showed statistically significant interactions: CFA15 x CFA20, CFA18 x CFA20, CFA2 x CFA15, CFA18 x CFA21, CFA2 x CFA18, CFA2 x CFA21, and CFA15 x CFA21 (Table 2).

**Table 2:** Pairwise tests for epistatic interaction among top GWAS markers.

Interaction	β3	STAT	p-value
CFA15 x CFA20	0.216	41.835	9.98 x 10^11^ *
CFA18 x CFA20	0.426	15.652	7.62 x 10^−5^ *
CFA2 x CFA15	-0.150	12.569	3.92 x 10^−4^ *
CFA18 x CFA21	-0.471	12.019	5.27 x 10^−4^ *
CFA2 x CFA18	-0.310	7.409	6.49 x 10^−3^*
CFA2 x CFA21	-0.098	5.815	1.59 x 10^−2^*
CFA15 x CFA21	-0.145	5.542	1.86 x 10^−2^*
CFA20 x CFA21	-0.066	2.459	1.17 x 10^−1^
CFA2 x CFA20	0.049	1.945	1.63 x 10^−1^
CFA15 x CFA18	-0.144	0.559	4.55 x 10^−1^

Interaction term coefficients (β3), test statistic, and p-value for each pair of the top five GWAS variants. Interactions with a p-value < 5 x 10^−2^ (marked with an asterisk) were considered statistically significant.

Two locus genotype and phenotype combinations for these variant pairs are shown in Fig 4B. The top CFA2 variant exhibits weak negative epistasis with the red-associated alleles at CFA15, 18, and 21 (Fig 4Bi). Two copies of the cream associated allele at the top CFA20 variant almost entirely masks the effect of the red-associated allele at the top CFA15 variant, and the top CFA15 variant exhibits negative epistasis with the top CFA21 variant (Fig 4Bii). The top CFA18 variant exhibits positive epistasis with the top CFA20 variant and negative epistasis with the top CFA21 variant (Fig 4Biii)

### A multilocus linear model predicts coat pheomelanin intensity with high accuracy

In agricultural, livestock, and canine genetics [50–53], a common approach for accurately predicting multigenic trait phenotypes such as body weight is to fit a statistical model with phenotype as a function of genotypes at multiple genetic markers. For traits with a significant genetic variance component, a model fit on a sufficiently large and representative training sample can be used to accurately predict phenotypes for new individuals given their genotypes without knowing the true underlying genetic architecture of the trait. The phenotypic predictions produced by these models can then be used to learn more about the genetic architecture of the trait. To assess the predictive value of our five associated loci and potential epistatic interactions, we fit a series of multiple linear regression models using genotype values at the top CFA2, 15, 18, 20, and 21 GWAS markers as independent variables.

First, we fit a model on normalized six point phenotype values that split the genotypes at all five loci into two variables each indicating whether or not they were heterozygous (“_1”), and whether or not they were homozygous for the red-associated allele (“_2”). The ratios of the model coefficients (β) for the _1 and _2 variables at each locus provided an additional evaluation of the dominance relationship between the two alleles: loci for which the _1 β was approximately half of the _2 β fit the assumption of additivity, whereas loci for which the _1 β was approximately zero were more consistent with the red-associated allele being recessive to the other allele, and loci for which the _1 and _2 βs were similar were more consistent with the red-associated allele being dominant to the other allele. Based on the β values for this model (Table 3), we concluded that the CFA2 and 20 loci explain more variance when coded as additive, the CFA15 locus explains more variance when the red-associated allele is coded as recessive, and the CFA18 and 21 loci explain more variance when the red-associated allele is coded as dominant. These findings broadly agree with our analysis of dominance effects at each locus shown in Fig 4.

**Table 3:** Evaluating additivity at top GWAS markers using linear model coefficients for heterozygotes versus red-associated allele homozygotes.

Variable	β	Std err	t-value	P > |t|	PRE
Intercept	-1.504	0.033	-45.080	<2.2x10^-16^	-
CFA2_1	0.472	0.029	16.016	<2.2x10^-16^	0.107
CFA_2	1.068	0.030	35.313	<2.2x10^-16^	0.369
CFA15_1	0.057	0.033	1.718	8.6x10^-2^	0.001
CFA15_2	0.208	0.032	6.499	<2.2x10^-16^	0.019
CFA18_1	0.234	0.049	4.759	<2.2x10^-16^	0.011
CFA18_2	0.208	0.082	2.542	1.1x10^-2^	0.003
CFA20_1	0.700	0.034	20.859	<2.2x10^-16^	0.169
CFA20_2	1.232	0.032	39.036	<2.2x10^-16^	0.417
CFA21_1	0.199	0.025	7.922	<2.2x10^-16^	0.029
CFA21_2	0.222	0.032	6.921	<2.2x10^-16^	0.022

Coefficients, coefficient standard error, t score values, and t test p-values for the y-intercept and each of the independent variables in a predictive model that encodes each dog’s genotype at each of the five top GWAS markers according to whether or not it was heterozygous (“_1”), and whether or not it was homozygous for the red-associated allele (“_2”). For each of the independent variables, the proportional reduction of error (PRE) value is also shown. PREs represent the fraction of the total sum of squares error that is accounted for by each independent variable.

Next, we fit five models with six point phenotype values as a function of genotype at each locus using its best dominance encoding in order to estimate the predictive power of each locus individually. This showed that the CFA2 and CFA20 loci each explained over 50% of the variance in six point phenotypes, while the CFA15, 18, and 21 loci each explained less than 10% of the variance (Table 4).

**Table 4:** Predictive power of individual loci.

Locus	Model	Ajd. R^2^	ln(likelihood)
CFA2	y = 2.364 + 1.435 * CFA2	0.501	-3,462.569
CFA15	y = 3.13 + 0.865 * CFA15_2	0.063	-4,142.560
CFA18	y = 3.448 + 1.413 * CFA18_red_dom	0.047	-4,160.124
CFA20	y = 1.603 + 1.512 * CFA20	0.508	-3,447.096
CFA18	y = 2.996 + 0.969 * CFA21_red_dom	0.077	-4,120.061

Best fit linear regression model equations, adjusted R-squared, and log likelihood scores are shown for each of the individual top GWAS SNPs using the dominance encoding most supported by the data in Table 3. The “CFA15_2” term encodes CFA15 genotype assuming that the red-associated allele is completely recessive, i.e. 1 if homozygous for the red-associated allele, and 0 if either of the other two genotype classes. The “CFA18_red_dom” and “CFA21_red_dom” terms encode CFA18 and CFA21 genotypes assuming that the red-associated allele is completely dominant, i.e. 1 if heterozygous or homozygous for the red-associated allele, and 0 if homozygous for the other allele.

To quantitatively determine the best combination of dominance encodings in a multilocus model, we fit 31 models with each possible combination of the additive and most likely dominance encoding at all five loci. A model treating all five loci as completely additive was able to explain 73% of variation in the six point phenotype (adjusted R-squared = 0.730) (Table 5A). The dominance model with the best fit (adjusted R-squared = 0.732) coded the red allele at CFA15 as recessive (“CFA15_2”), the red alleles at CFA18 and CFA21 as dominant (“CFA18_red_dom”, “CFA21_red_dom”), and CFA2 and CFA20 as additive (Table 5B).

**Table 5:** Comparison of multilocus coat pheomelanin intensity predictive models.

	Variables	β ± se	t-value	P>|t|	PRE	Adj. R^2^	ln(likelihood)
**A.**	Intercept	1.012 ± 0.049	20.831	<2.2x10^-16^	-	0.7300	-2,795.30
	CFA2	0.915 ± 0.026	35.074	<2.2x10^-16^	0.365		
	CFA15	0.191 ± 0.026	7.225	<2.2x10^-16^	0.024		
	CFA18	0.272 ± 0.056	4.85	<2.2x10^-16^	0.011		
	CFA20	1.038 ± 0.026	39.262	<2.2x10^-16^	0.419		
	CFA21	0.215 ± 0.027	0.027	<2.2x10^-16^	0.029		
**B.**	Intercept	1.074 ± 0.043	25.088	<2.2x10^-16^	-	0.7324	-2,785.92
	CFA2	0.920 ± 0.026	35.666	<2.2x10^-16^	0.373		
	CFA15_2	0.286 ± 0.039	7.256	<2.2x10^-16^	0.024		
	CFA18_red_dom	0.405 ± 0.074	5.444	<2.2x10^-16^	0.014		
	CFA20	1.037 ± 0.026	39.453	<2.2x10^-16^	0.421		
	CFA21_red_dom	0.355 ± 0.040	8.904	<2.2x10^-16^	0.036		
**C.**	Intercept	1.606 ± 0.062	25.834	<2.2x10^-16^	-	0.5394	-3375.46
	CFA15_2	0.053 ± 0.096	0.550	5.82 x 10^−1^	0.000		
	CFA15_2 x CFA20	0.374 ± 0.063	5.956	<2.2x10^-16^	0.016		
	CFA20	1.290 ± 0.043	29.844	<2.2x10^-16^	0.294		
**D.**	Intercept	1.095 ± 0.054	20.250	<2.2x10^-16^	-	0.7353	-2772.11
	CFA2	0.908 ± 0.026	35.087	<2.2x10^-16^	0.366		
	CFA15_2	0.167 ± 0.081	2.050	4.1 x 10^−2^	0.002		
	CFA15_2 x CFA20	0.161 ± 0.049	3.291	1.0 x 10^−3^	0.005		
	CFA15_2 x CFA21_red_dom	-0.139 ± 0.079	-1.752	8.0 x 10^−2^	0.001		
	CFA18_red_dom	1.225 ± 0.217	5.65	<2.2x10^-16^	0.015		
	CFA18_red_dom: CFA20	-0.381 ± 0.112	-3.400	1.0 x 10^−3^	0.005		
	CFA18_red_dom: CFA21_red_dom	-0.308 ± 0.174	-1.772	7.7 x 10^−2^	0.001		
	CFA20	0.985 ± 0.034	28.85	<2.2x10^-16^	0.281		
	CFA21_red_dom	0.436 ± 0.055	7.944	<2.2x10^-16^	0.029		
**E.**	Intercept	1.134 ± 0.051	22.195	<2.2x10^-16^	-	0.7346	-2,775.53
	CFA2	0.908 ± 0.026	35.043	<2.2x10^-16^	0.365		
	CFA15_2	0.102 ± 0.073	1.387	1.67x10^-1^	0.001		
	CFA15_2 x CFA20	0.148 ± 0.048	3.061	2.0x10^-3^	0.004		
	CFA18_red_dom	1.017 ± 0.185	5.496	<2.2x10^-16^	0.014		
	CFA18_red_dom x CFA20	-0.406 ± 0.112	-3.640	<2.2x10^-16^	0.006		
	CFA20	0.992± 0.034	29.141	<2.2x10^-16^	0.285		

Coefficients, coefficient standard error, t score values, t test p-values, and PRE for the y-intercept and each of the independent variables in the best fit linear model incorporating non-additivity and pairwise epistasis. Section **A.** shows the base model that assumes perfect additivity at each locus and no interactions between loci. Section **B.** shows the best fit model incorporating dominance at all five loci. Section **C.** shows a model consisting of only the two previously reported loci (CFA15 and CFA20) using their best dominance encoding, and their pairwise interaction (CFA15_2 x CFA20). Section **D.** shows the best fit model incorporating both the dominance terms in model B. and two pairwise epistasis terms: CFA15_2 x CFA20 and CFA18_red_dom x CFA20. Section **E.** shows a reduced version of model D. that only includes terms that explained > 0.1% of variance (PRE > 1 x 10^−3^) in model D. and shows similar performance.

**Fig 5:**
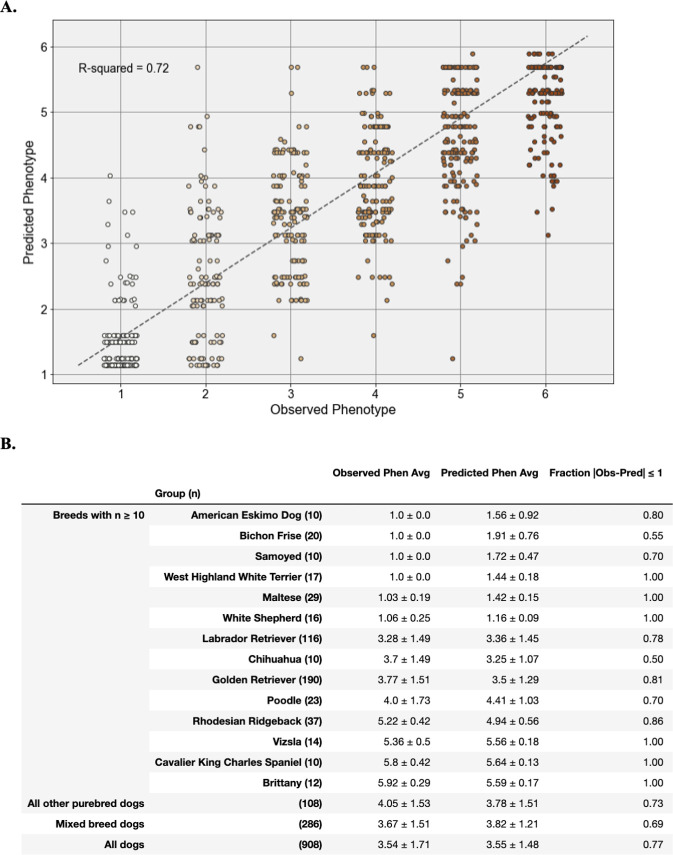
Performance of the best fit multivariate linear regression model for pheomelanin intensity phenotypes in validation cohort. **A.** Strip plot of observed versus predicted phenotypes for all dogs in the validation dataset using the predictive model shown in Table 3. The adjusted R-squared value is shown in the top right hand corner. Each point represents a single dog, colored according to its observed six point phenotype. **B.** Performance of the multivariate linear regression model within and across breeds. For each row, observed and predicted phenotype averages are shown ± their standard deviation. To assess model prediction accuracy in each breed or group, each row shows the fraction of dogs with a predicted phenotype value within one point of their observed phenotype (on the six point phenotype scale).

Next, we fit 4,095 models with each possible combination of the seven statistically significant pairwise epistatic interactions and the five loci in the best fit dominance model (S1 File). A model using the best dominance encodings for only the two previously reported loci—CFA15_2 and CFA20—and their pairwise interaction explained 54% of variance (Adjusted R-squared = 0.5394) (Table 5C). The model with the highest adjusted R-squared value (0.7353) included terms for each of the five loci in the best fit dominance model as well as interaction terms for CFA15_2 x CFA20, CFA15_2 x CFA21, CFA18_red_dom x CFA20, and CFA18_red_dom x CFA21_red_dom (Table 5D). However, three terms accounted for less than 1% the total variance each: CFA15_2, CFA15_2 x CFA21, and CFA18_red_dom x CFA21_red_dom. A reduced model excluding these terms (Table 5E) was not significantly less predictive than the full best fit model (Table 5D) (likelihood ratio test p-value = 7.70 x 10^−2^) and was significantly more predictive than either the purely additive model (Table 5A) (likelihood ratio test p-value = 2.595 x 10^−9^) or the model with the best fit dominance encoding and no epistasis (Table 5B) (likelihood ratio test p-value = 5.104 x 10^−6^). We applied the reduced best fit predictive model to the 908 dogs in the validation sample and found that it was able to explain 72% (adjusted R-squared = 0.7211) of variation in coat pheomelanin intensity across all dogs (Fig 5A).

In order to evaluate the model’s performance in specific breeds, some of which had insufficient sample sizes or phenotypic variation to calculate a meaningful R-squared value, we also calculated the percentage of dogs in a breed for which the model predicted a phenotype value within 1 point of the observed phenotype value (Fig 5B). This value was 77% across all validation dogs, and 69% across mixed breed validation dogs. Among purebred validation dogs, the model’s performance was generally high in breeds that are fixed for a narrow range of coat pheomelanin intensity (e.g. Samoyeds and Irish Setters) and lower in breeds with a wide range of coat colors (e.g. Chihuahuas and Poodles). Some notable exceptions to this pattern were Bichon Frisé, which are fixed for cream or white coats but poorly predicted by this model, and Golden Retrievers and Yellow Labrador retrievers, which display nearly the full range of coat pheomelanin intensity variation and for which our model is highly predictive.

## Discussion

Our understanding of the genetic basis of variable pheomelanin intensity in dog coat color has progressed recently with the discovery of associations between this phenotype and three genes: *MC1R*, *MFSD12*, and *KITLG* [18, 19, 32]. However, the entire genetic architecture of this apparently multigenic phenotype remains obscure because the explanatory power of known variants in/near these genes is mostly limited to a small number of breeds. Here we have shown that the hypothetical “I locus” controlling coat pheomelanin intensity variation actually maps to at least five separate genetic loci that together explain the majority of phenotypic variation in purebred and mixed breed dogs, including several breeds with highly variable coat pheomelanin intensity.

The top CFA2 variant falls within a long intergenic non-coding RNA (lincRNA) with unknown functional significance in domestic dog. Many mammalian (including dog) lincRNAs are known to modulate the expression of nearby protein-coding genes via *cis-*regulatory mechanisms [54–57]. The closest annotated canine protein-coding gene is RUNX family transcription factor 3 (*RUNX3*), located approximately 82 kb downstream of ENSCAFG00000042716 at CFA2: 74,829,960–74,856,947. *RUNX3* encodes a transcription factor that shows reduced expression in hair follicles in human premature hair greying and appears to regulate expression of several other genes that also show reduced expression in premature greying samples [58]. *RUNX3* is also known to be a regulator of hair shape determination during murine embryonic development [59]. We therefore suggest that the CFA2 locus identified in our GWAS may be tagging a *cis*-regulatory module consisting of ENSCAFG00000042716, *RUNX3*, and possibly other unknown genic variants or functional genomic elements. Identifying the causal mutations underlying this association will require fine mapping of the locus, as well as molecular experiments to directly assess the functional impacts of any candidate mutations.

The top CFA21 variant is an intronic substitution in the *TYR* gene. This gene encodes the enzyme tyrosinase, which catalyzes the oxidation of l-dihydroxy-phenylalanine (DOPA) to DOPA quinone, a precursor of both eumelanin and pheomelanin. Mutations in and around *TYR* produce varying degrees of pheomelanin dilution in several mammalian species by decreasing the amount of pheomelanin produced in hair shaft melanosomes [22–31]. Canine geneticists have previously hypothesized that *TYR* mutations might also produce pheomelanin dilution in dogs [60], but earlier candidate-gene studies of exonic variants in the gene did not uncover any associated variants [21]. However, the hypothesis that *TYR* variants can modulate coat pheomelanin intensity in dogs was finally supported when a recent study identified a missense mutation in the *TYR* gene as causal for a unique temperature-dependent pigment dilution phenotype (acromelanism) in a single dog [61]. Our study further solidifies this hypothesis and provides the first documented link between canine *TYR* variants and non temperature-dependent coat pheomelanin intensity variation, although fine mapping and functional validation will be required to definitively identify a causal variant. In multiple species, some of the genes located nearby *TYR* on CFA21 (including *NOX4* [62] and *GRM5* [63, 64]) are also known to be involved in skin pigmentation, so it is also possible that other variants outside of the *TYR* gene may be driving or contributing to the association signal on CFA21.

The connection between coat pheomelanin intensity and the gene tagged by the top CFA18 association is less apparent. The A to G substitution at this variant results in an amino acid substitution from isoleucine to methionine in the solute carrier family 26 member 4 (SLC26A4) protein. Based on computational modeling (Sorting Intolerant from Tolerant (SIFT) score = 0.03), this substitution is predicted to be somewhat deleterious [65]. However, its functional consequences in dogs have not been reported. While the *SLC26A4* gene has no clear connection to hair coat pigmentation in mammals, it does play a role in a variety of hearing impairment phenotypes in human and inner ear abnormalities in mouse, including hyperpigmentation in the stria vascularis [66] and degeneration of inner ear hair cells [67]. There is substantial precedent for genes that affect inner ear function also affecting canine coat color: certain mutations in and around the microphthalmia-associated transcription factor (*MITF*) [13] and *PMEL* (also known as *SILV*) [10] genes, which are responsible for the piebald and merle coat patterns (respectively), cause varying degrees of deafness due to insufficient pigment expression in specialized hairs in the inner ear [10, 68]. Additionally, mutations in and around *KITLG* cause hearing loss in humans [69]. Due to its low minor allele frequency in our dataset (5%), the top CFA18 GWAS marker only explains 4% of variance in the intensity phenotype across all dogs, but still has a significant effect size both the GWAS and the predictive model. It is most variable in purebred Poodles, where it has a minor allele frequency of 46% (Fig 3B). This association will require additional validation, ideally in a larger panel of purebred Poodles.

We also found significant evidence for epistatic interactions between the CFA20 locus and both the CFA15 and CFA18 loci. In fact, based on the PRE values in our linear regression analysis, the effect of the CFA15 x CFA20 interaction is greater than the effect of the top CFA15 variant (Table 5C–5E). Based on what is currently known about the molecular functions of the three genes closest to these variants, it is unclear exactly how these epistatic relationships might arise: The *KITLG* gene on CFA15 encodes a ligand that binds to the c-Kit protein on the surface of melanocytes, triggering the Ras/MAPK signaling pathway and stimulating melanocyte proliferation and melanogenesis [70–72]. The CFA15 CNV that our GWAS signal appears to be tagging falls upstream of the dog *KITLG* coding sequence, indicating that its likely affecting pheomelanin intensity by modulating *KITLG* expression. As noted in the study that first reported this association [32], this assertion is supported by the fact that genetic variants that alter the expression of *KITLG* have been associated with both pheomelanin and eumelanin dilution in several mammalian species [71, 73–77]. The *SLC26A4* gene on CFA18 encodes a transmembrane ion transporter that is highly expressed on the apical surfaces of epithelial cells in the inner ear [78], thyroid [79], and kidney [80] in humans and mice. As mentioned above, mutations in *SLC26A4* have been associated with abnormal melanin deposition and hair cell degeneration in the inner ear. Unfortunately, little is known about the role that *SLC26A4* plays in these phenotypes. It is also possible that our GWAS signal on CFA18 is actually driven by some other nearby gene that happens to be in high linkage disequilibrium with our top CFA18 variant in this study sample. The *MFSD12* gene on CFA20 encodes a transmembrane solute transporter that localizes to melanocyte lysosomes and/or late endosomes in mice [81]. The molecular mechanism by which *MFSD12* influences hair pigmentation is still not well understood, but it has been suggested that it might regulate melanosome autophagy [81]. If this is the case, then it is possible that the *MFSD12* cream-associated variant masks the effect of the *KITLG* red-associated variant by causing abnormal degradation of melanosomes downstream of pro-melanogenic signaling by *KITLG*.

A multigenic predictive model using genotypes at the most strongly associated single-nucleotide genetic markers on CFA2, 15, 18, 20, and 21, plus two interaction terms, was able to explain over 70% of the phenotypic variation across both the GWAS cohort and an independent validation cohort containing individuals from over 60 breeds as well as mixed breed dogs. This represents a gain of approximately 20% variance explained compared to a model using only the two previously discovered loci (Table 5C). Because coat pheomelanin intensity appears to be a truly continuous phenotype across dogs, it is likely that the remaining variation is controlled by multiple additional loci. Currently, the only other known canine pheomelanin intensity loci are two highly breed-specific mutations in the *MC1R* gene, which underlie cream coats in Siberian Huskies and Australian Cattle Dogs [18]. These variants were not typed on our genotyping array, so we were unable to include them in our analyses. We also note that our study did not incorporate the progressive “fading” phenotype seen in several dog breeds—most notably Poodles—in which coat pigmentation lightens as a dog reaches adulthood. It is unclear if and to what extent this hypothetical dominant trait affects or interacts with pheomelanin intensity. The fading phenotypes of dogs in our study are unknown, but future studies may reveal connections between progressive fading and coat pheomelanin intensity variation.

Taken together, these results demonstrate that coat pheomelanin intensity in the domestic dog is a multigenic trait both across and within breeds, and that some loci controlling this trait likely interact via unknown biological pathways. Further fine mapping and experimental investigation will be required to validate the three novel associations, to characterize the roles these and other genetic loci play in pigmentation in dogs and other species, and to determine whether any mutations associated with coat pheomelanin intensity variation also exhibit pleiotropic effects on canine health, such as deafness.

## Supporting information

S1 Appendix (PDF)

S1 Table (TSV)

S2 Table (XLSX)

S3 Table (TSV)

S4 Table (XLSX)

S1 File (TXT)
