# Supplementary material for: Five genetic variants explain over 70% of hair coat pheomelanin intensity variation in purebred and mixed breed domestic dogs
Source: PLoS One. 2021 May 27;16(5):e0250579. doi: 10.1371/journal.pone.0250579 (PMC8158882; doi:10.1371/journal.pone.0250579)
Supplement: S1 Appendix — (PDF) [file pone.0250579.s001.pdf]

S1 Appendix

S1 Fig. Phenotyping validation on 350 randomly selected dogs

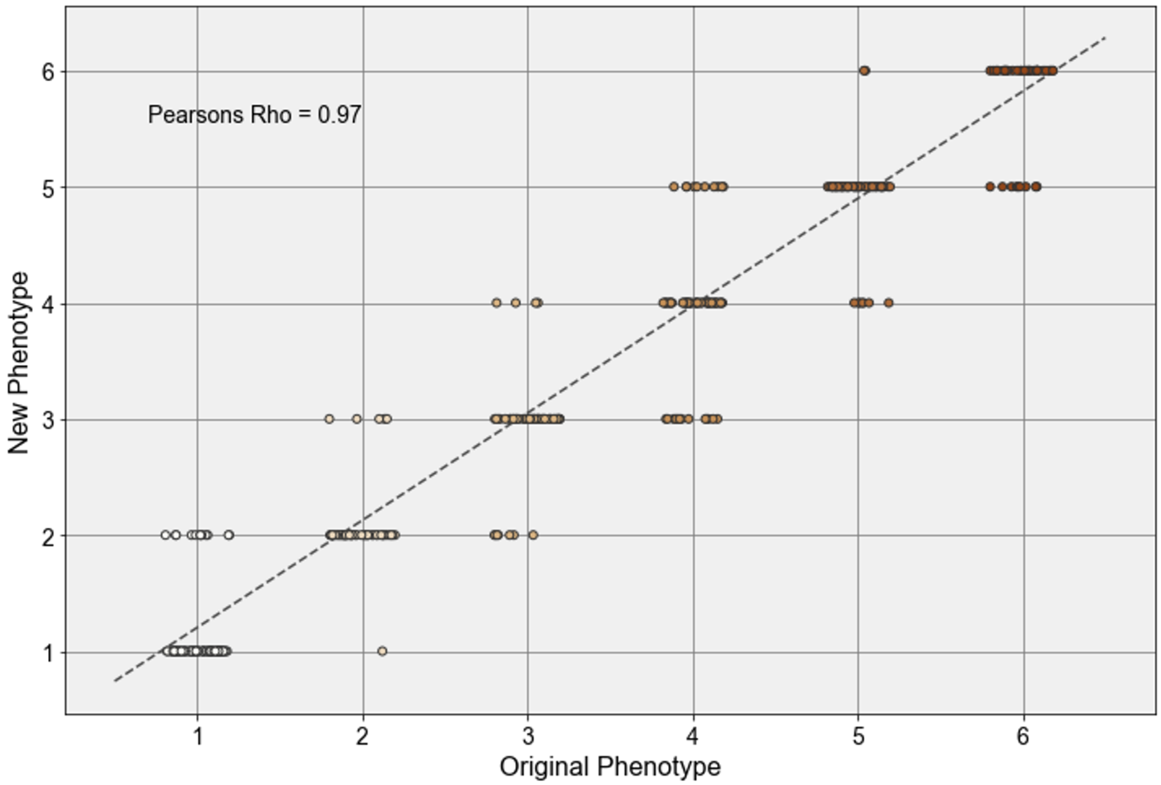

S1 Fig. Phenotyping validation on 350 randomly selected dogs: Strip plot showing original versus re-scored 6 point phenotypes for a random sample of 350 dogs from the discovery sample. The correlation coefficient (Pearson's Rho) between the original and new phenotype scores is shown in the upper left hand corner of the plot.

S2 Fig. Manhattan plots for additional GWAS

A. 6pt phenotype, no covariates

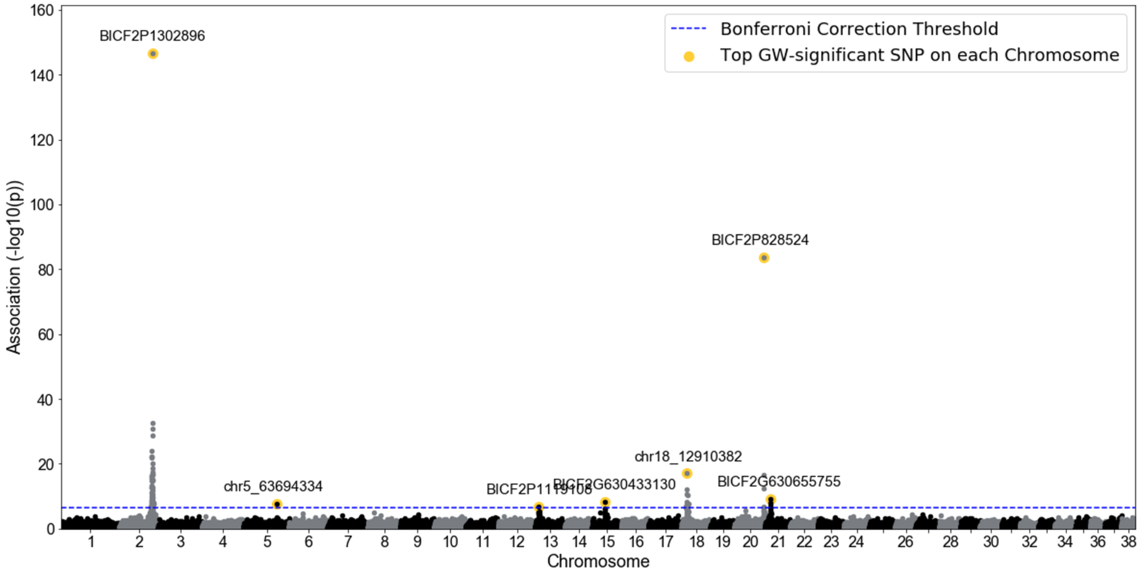

B. Binary phenotype, with covariates

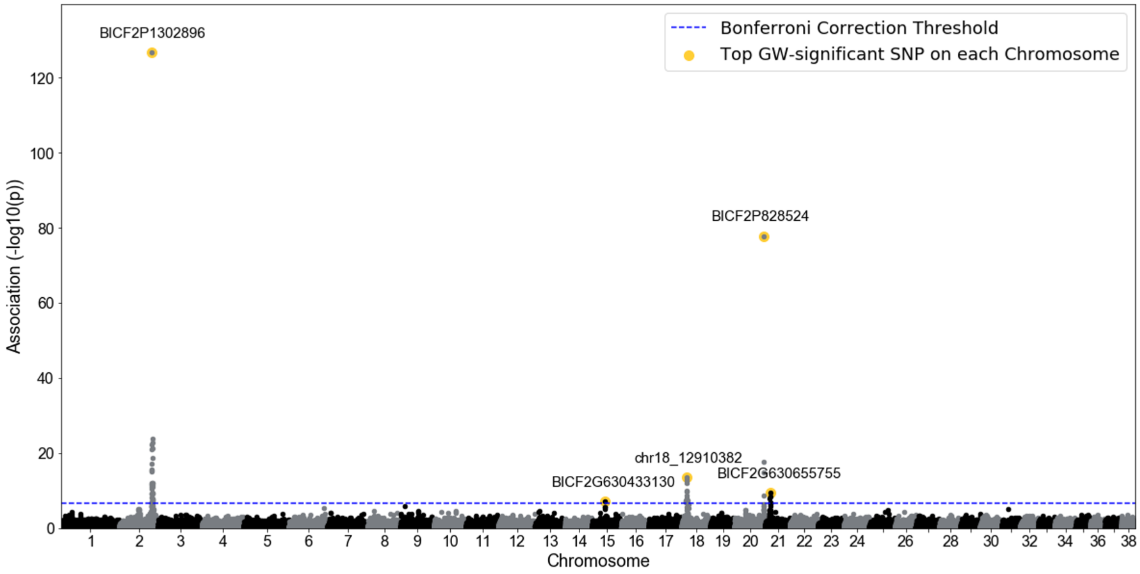

C. Binary phenotype, no covariates

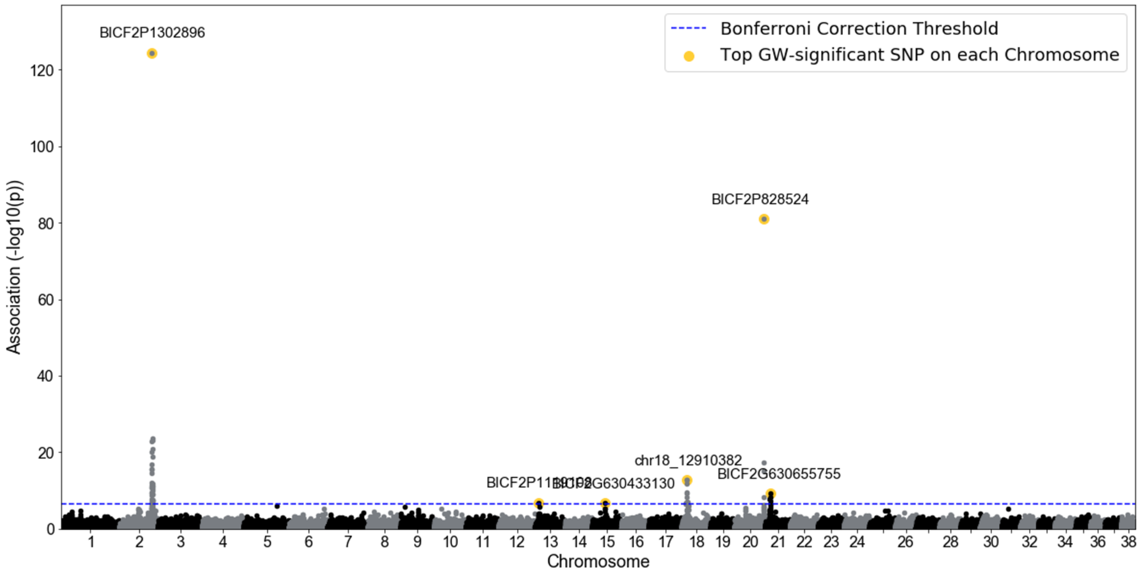

S3 Fig. Detailed view of regions surrounding top GWAS SNPs

A.

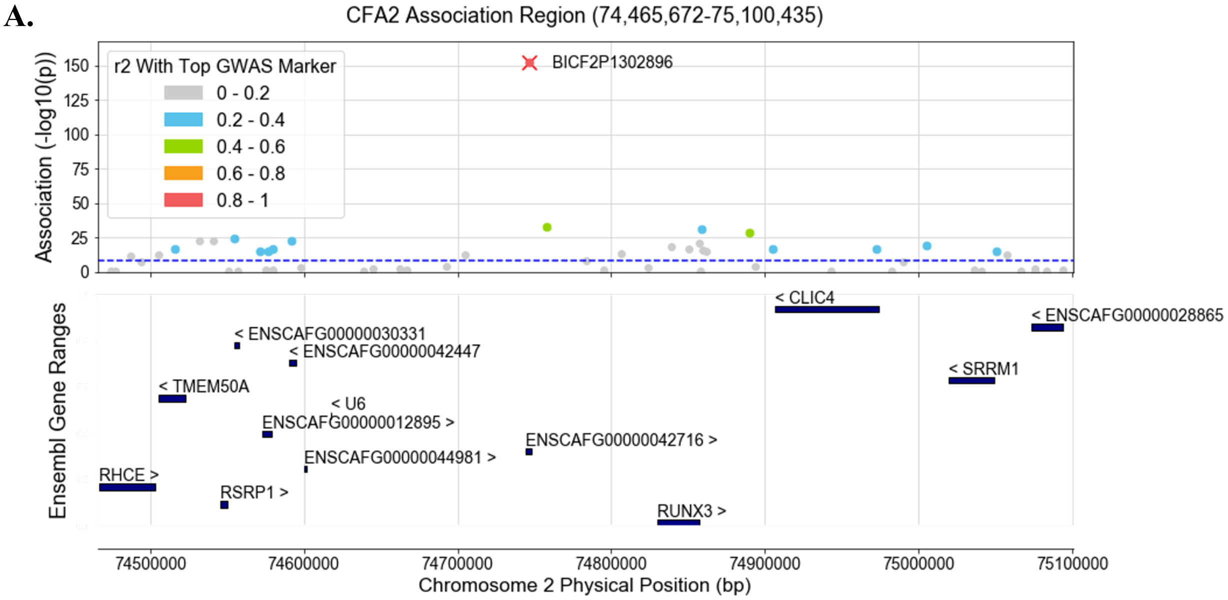

B.

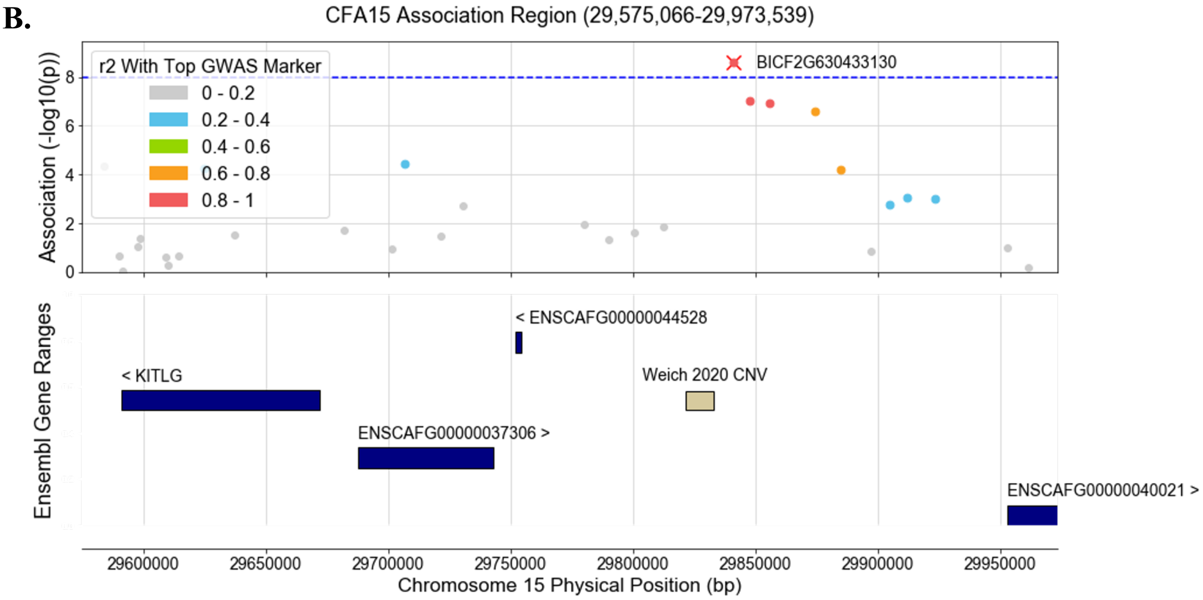

C.

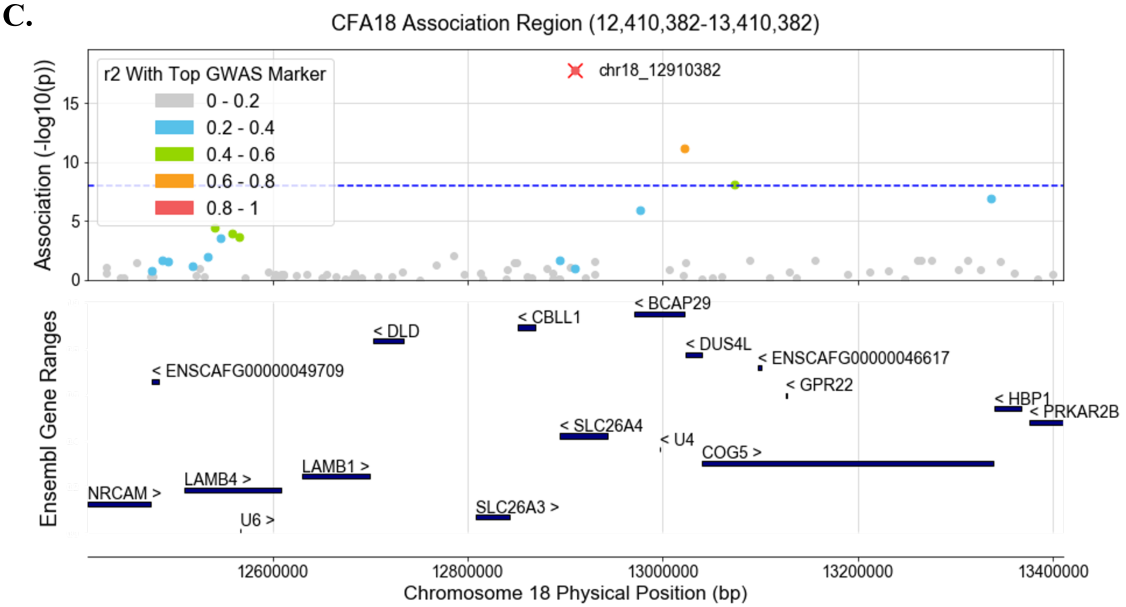

D.

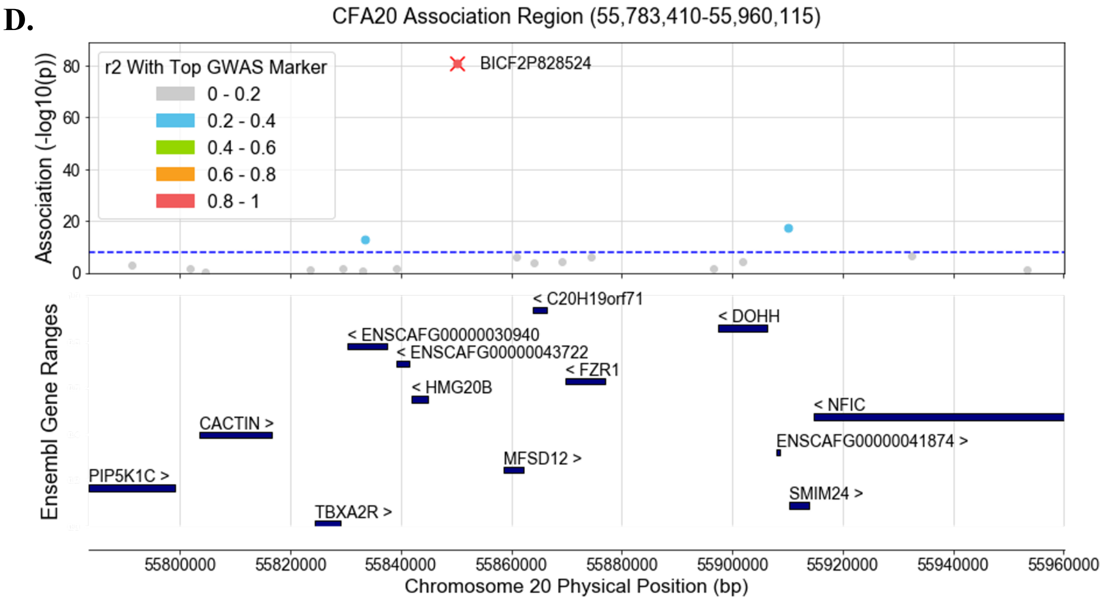

E.

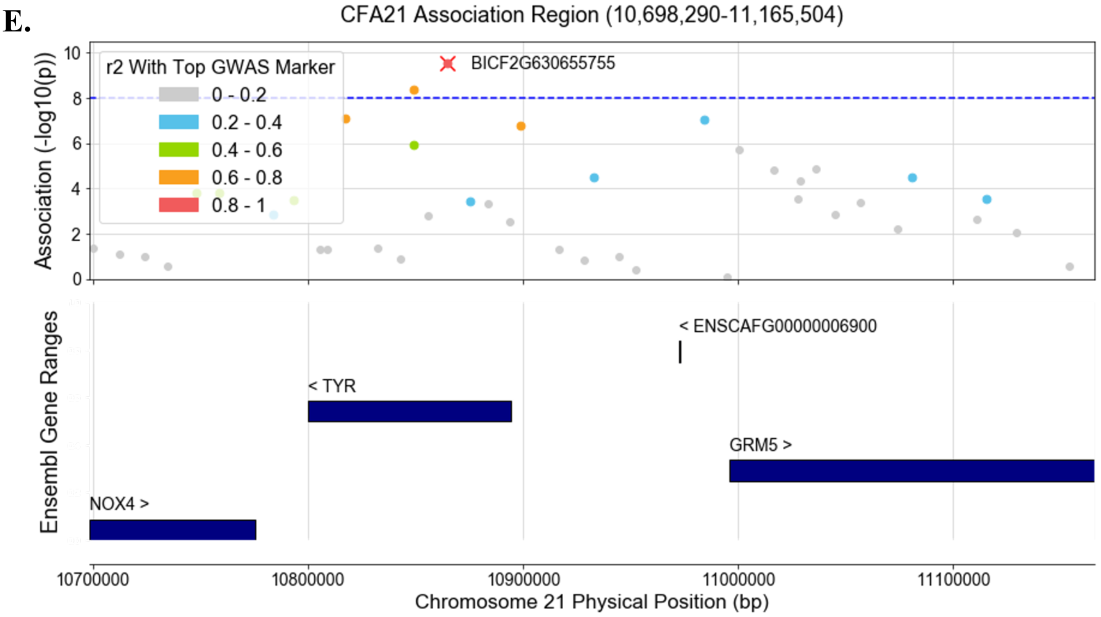

S3 Fig. Detailed view of regions surrounding the top CFA2 (A), 15 (B), 18 (C), 20 (D), and 21 (e) GWAS markers. Each panel shows the genomic region defined by the positions of the first upstream marker and last downstream marker with $r^2 \geq 0.2$ with the most significant GWAS marker on the chromosome (indicated by a red "x"). The top panel of each figure shows the GWAS -log10(p-value) and physical position of all GWAS markers in the region, colored by their r^2 with the top GWAS marker. The bottom panel of each figure shows the canFam3.1 locations of known dog transcripts in this region. Transcription ranges are shown as dark blue rectangles, each of which is labelled with its Ensembl Genes (version 95) [81] transcript or gene name and its strand orientation (">" = plus strand, "<" = minus strand).

S4 Fig. CFA15 top marker genotype correlates with sequencing coverage in known CNV

A.

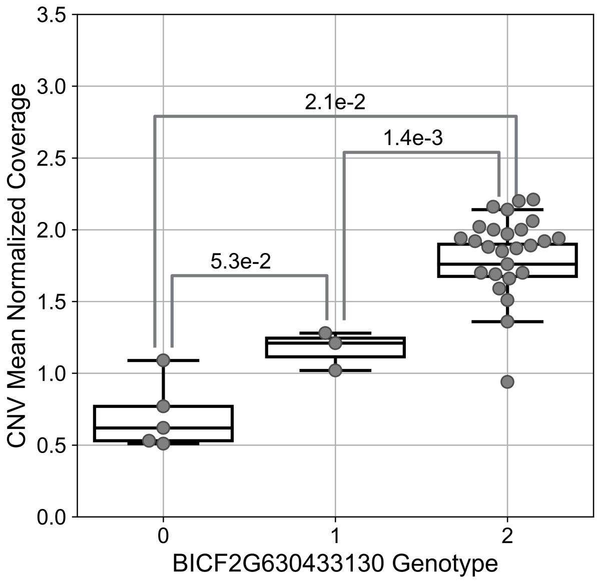

B.

SRA Run ID	Sample Name	Breed	BICF2G630433130 Genotype	CNV Read Depth
ERR2196100	ERR2196100	Bichon Frisé	0	0.51
SRR7120175	152721_S5	Labrador Retriever	0	0.77
SRR7107894	SRR7107894	Golden Retriever	0	0.53
SRR7120211	SRR7120211	Samoyed	0	1.09
SRR2827573	SRR2827573	Samoyed	0	0.62
SRR7120163	173006_S10	Golden Retriever	1	1.02
SRR7120174	149323_S6	Labrador Retriever	1	1.28
ERR2196098	ERR2196098	Bichon Frisé	1	1.21
SRR8614086	SRR8614086	Irish Setter	2	1.94
SRR1784129	SRR1784129	NSDTR	2	0.94
SRR7107577	SRR7107577	Vizsla	2	1.59
SRR7120182	SRR7120182	Labrador Retriever	2	1.69
SRR7120161	SRR7120161	Golden Retriever	2	1.85
SRR2094390	BrittanySpaniel01	Brittany	2	1.66
SRR8614059	SRR8614059	Irish Setter	2	1.87
ERR2196099	ERR2196099	Bichon Frisé	2	1.70
SRR7120176	160822_S4	Labrador Retriever	2	1.76
SRR7120167	IrishSetter01	Irish Setter	2	2.14
SRR7120160	140447_S11	Golden Retriever	2	2.02
SRR7107980	CFA.117999	Labrador Retriever	2	1.70
SRR7107926	GoldenRetriever13	Golden Retriever	2	1.36
ERR2196101	ERR2196101	Bichon Frisé	2	1.92
SRR8614027	SRR8614027	Irish Setter	2	1.88

S4 Fig. CFA15 top marker genotype correlates with sequencing coverage in known CNV: A. Boxplots overlaid with strip plots show the distribution of mean normalized depth of coverage across the CFA15 CNV characterized in Weich et al. 2020 [32] (CFA15: 29,821,450-29,832,950 bp) for dogs with each possible BICF2G630433130 genotype. Each point represents a single dog. Kruskal Wallis test p-values are shown for each pair of genotypes. **B.** SRA run ID and sample name, breed, BICF2G630433130 genotype (coded as number of red-associated alleles), and CFA15 CNV mean normalized depth of coverage for all dogs shown in A.
